# A Case Study of a Hailstorm in the Shanghai Region: Leveraging Multisource Observational Data and a Novel Single-Polarization X-Band Array Weather Radar

**DOI:** 10.3390/s25092870

**Published:** 2025-05-01

**Authors:** Xiaoqiong Zhen, Hongbin Chen, Hongrong Shi, Xuehua Fan, Haojun Chen, Jie Fu, Wanyi Wei, Shuqing Ma, Ling Yang, Jianxin He

**Affiliations:** 1 College of Electronic Engineering, Chengdu University of Information Technology, Chengdu 610225, China; zhenxq@cuit.edu.cn (X.Z.); 3220301005@stu.cuit.edu.cn (J.F.); cimyang@cuit.edu.cn (L.Y.); hjx@cuit.edu.cn (J.H.); 2 Key Laboratory of Middle Atmosphere and Global Environment Observation, Institute of Atmospheric Physics, Chinese Academy of Sciences, Beijing 100029, China; shihrong@mail.iap.ac.cn (H.S.); fxh@mail.iap.ac.cn (X.F.); 3 College of Earth and Planetary Sciences, University of Chinese Academy of Sciences, Beijing 100049, China; 4 Shanghai Meteorological Information and Technology Support Center, Shanghai 200030, China; chj513@163.com; 5 Eastone Washon Science and Technology Ltd., Shaoxing 312000, China; weiwanyi@wholesenseradar.com; 6 Meteorological Observation Center of China Meteorological Administration, Beijing 100081, China; msqaoc@cma.gov.cn

**Keywords:** hailstorm precipitation, novel X-band AWR data, multisource observational data, divergence, vertical vorticity component

## Abstract

This study investigates a severe summer convective hailstorm that occurred in Shanghai on 18 August 2019, using multisource meteorological datasets, with a particular focus on the innovative application of a single-polarization X-band array weather radar (AWR). Radiosonde data revealed high convective available potential energy and unstable atmospheric indices, while wind profiler radars (WPRs) showed initial easterly moisture transport near the ground and strong southwesterly flow aloft, both contributing significantly to intense convection. Ground-based automatic meteorological stations (AMSs) recorded abrupt temperature drops of approximately 10 °C and wind speed increases exceeding 20 m s^−1^, which aligned closely with the rapid expansion of the hailstorm. In addition, an integrated analysis of data from AWR, WPRs, and AMSs enabled detailed tracking of the storm’s evolution, providing deeper insights into the interplay between moisture transport and dynamic lifting. The AWR’s unique ability to capture divergence and vorticity fields at different altitudes revealed low-level convergence coupled with high-level divergence and cyclonic rotation, sustaining convective updrafts. This study underscores the value of high-resolution AWR data in capturing short-lived, intense precipitation processes, thereby enhancing our understanding of wind field structures and storm development. These findings highlight the comprehensive application of AWR data and the potential of this new high-spatiotemporal-resolution radar for investigating the mechanisms of short-lived severe convective processes.

## 1. Introduction

Hail is a common type of severe weather phenomenon, typically accompanying strong convective precipitation events. It is characterized by locality, sudden onset, and transient duration. Hailstorms can cause devastating damage to agricultural crops, leading to direct economic losses, particularly when occurring during the growth period. According to national agricultural meteorological disaster statistics from 1978 to 2016 [1], wind and hail disasters rank third among major meteorological hazards in China, affecting an average annual area of approximately 4.47 million hectares and resulting in economic losses of about CNY 112.1 billion. Beyond agriculture, hailstorms can significantly impact livestock farming, transportation, and infrastructure, even posing threats to human life.

In the detection of convective cloud precipitation, weather radar, Doppler weather radar, and subsequent dual-polarization upgrades have become widely recognized tools in meteorological observation, both domestically and internationally [2]. Leveraging the detection advantages of weather radar, meteorologists and researchers have extensively studied convective clouds [2,3,4,5,6,7,8,9,10]. Despite extensive radar networks such as NEXRAD (Next-Generation Weather Radar), MPAR (Multifunction Phased-Array Radar), and TDWR (Terminal Doppler Weather Radar) in the United States; OPERA in Europe; and CINRAD in China, storms with small scales and short lifecycles place higher demands on radar detection capabilities [3,11,12,13,14]. Consequently, developing radars with rapid scanning capabilities and exploring optimized scanning modes to enhance the spatiotemporal resolution of radar data have become crucial research directions in weather radar development [15,16,17,18].

Employing phased-array antenna technology is an effective approach to achieving rapid scanning capabilities in weather radars. Indeed, phased-array technology has already been applied in the weather radar domain and has produced valuable research outcomes in hailstorm detection and analysis [19,20,21,22,23,24,25,26,27,28,29,30,31]. On 15 August 2006, the U.S. National Weather Radar Testbed Phased-Array Radar (NWRT PAR) observed a hail event using a 28 s volume scanning interval. High-resolution NWRT data revealed a threefold increase in the area of reflectivity factors exceeding 65 dBZ within 26 s and a rapid collapse of the precipitation core [25]. Emersic et al. analyzed combined NWRT and lightning mapping array (LMA) observations, demonstrating a correlation between lightning activity and storm kinematics during hail formation. The findings indicated that lightning rates were associated with the wet growth of hailstones [32]. To assess the impact of high-temporal-resolution radar data on forecasters’ warning decision-making processes, the United States conducted the Phased-Array Radar Innovative Sensing Experiment (PARISE) in 2013. A comparison of high-spatiotemporal-resolution NWRT data (updated every 1 min) with conventional radar data (updated every 5 min) showed that the high-resolution data group had a lower false alarm rate, earlier identification of storm precursors, and more accurate assessments of potential threats from severe storms, thus confirming the advantages of high-resolution radar data in severe weather warning decisions [33,34]. In 2005, a military strategic radar was converted into the Meteorological Weather Radar 2005 X-band Phased Array (MWR-05XP). In May 2007, MWR-05XP captured a squall line event producing 3.8 cm hailstones and gusts of up to 34 ms^−1^, accompanied by radar echo gaps at greater distances. On another day in May, the MWR-05XP detected thin reflectivity factor lines at lower levels within the storm, eventually generating 2.5 cm hailstones. In June 2008, during a supercell storm, the radar observed no vortex signatures within the precipitation cell, yet the storm produced tennis-ball-sized hailstones [28].

With the increasing application of PAR technology in meteorology, China has progressively developed and field-tested PAWR [35,36,37,38,39]. In March 2021, an X-band dual-polarization PAR (APAR) in Chengdu successfully detected a local hailstorm. Data analyses demonstrated APAR’s ability to finely resolve the vertical structures of hail clouds and capture dynamic changes during hailstone descent, such as increases in differential reflectivity (Z_DR_) during melting. Unlike typical summer hailstorms, this event featured convective echo tops of around only 5 km, without common features like three-body scattering, V-shaped notches, hook echoes, or lightning activity [40,41]. In June 2020, a convective hailstorm event at Beijing Daxing International Airport was captured by a C-band PAR, showcasing clearer velocity vortex structures and richer reflectivity layers than those of operational S-band SA radar. These advantages underscore C-PAR’s superior spatiotemporal resolution and suitability for hailstorm detection [42]. These studies collectively indicate that PARs, irrespective of their polarization capabilities, provide high-resolution data critical for radar network observations, wind field retrieval in overlapping detection areas, and detailed analyses of storm dynamics, enhancing our understanding of short-duration convective precipitation and hail formation mechanisms.

Inspired by networked weather radars and motivated by meteorologists’ and atmospheric science researchers’ growing demand for high spatiotemporal resolution radar data, in 2015, the China Meteorological Administration Meteorological Observation Center proposed and designed an array weather radar (AWR), an X-band one-dimensional phased-array radar system capable of accommodating multiple phased-array radar frontends. In collaboration with radar manufacturers, the first AWR system with three phased-array radar frontends was developed in 2017, demonstrating its observational capabilities and the utility of high-resolution radar data through field experiments [43,44,45,46,47]. Compared with conventional networked phased-array weather radar systems [10,14,19,21,48], each AWR system typically comprises at least three phased-array radar frontends, usually arranged in a triangular configuration and employing a unique synchronized azimuthal scanning (SAS) strategy to minimize the detection data time difference (DTD) between data collected by each radar frontend. The three radar frontends form overlapped observation areas, such as enhanced detection areas (EDAs) and a fine detection area (FDA), and the most distinctive advantage of the AWR is its ability to provide high-spatiotemporal-resolution wind field data within storm cells located in EDAs and FDAs. Unlike conventional phased-array radar networks, the multiple phased-array radar frontends in the AWR system offer superior data consistency, as these radar frontends are controlled by a single radar backend. Consequently, the coordinated control among multiple radar frontends is simplified, enabling the implementation of the system’s distinctive SAS strategy [47].

On 18 August 2019, a severe convective event accompanied by hail occurred in the Shanghai region. From 06:00:00 to 07:30:00 UTC, a single-polarization X-band AWR, equipped with three phased-array radar frontends (referred to hereafter as the Shanghai AWR) detected this strong precipitation event. Other observation equipment also captured this precipitation event, including an S-band CINRAD radar (referred to as the Qingpu SA), two wind profiler radars (WPRs), a radiosonde from Baoshan National Climate Observatory, and two ground-based automatic meteorological stations (AMSs). This study focuses on analyzing the storm’s evolution and variations in relevant physical products as the intense precipitation entered overlapped observation areas of the Shanghai AWR.

To completely leverage the advantages of multisource meteorological observational data, a comprehensive analysis of the precipitation event was conducted using data from various meteorological equipment throughout its lifecycle. Among the multisource observational datasets, the uniquely distinctive data are provided by the Shanghai AWR, including reflectivity factors, horizontal divergence fields, and vertical vorticity component fields at multiple altitudes, characterized by their high spatiotemporal resolution. The contents of each section are summarized as follows: Section 2.1 presents the storm warning information and the layout of observational equipment. Section 2.2 provides synoptic analysis and the meteorological background. Section 3.1 analyzes data from the WPRs and ground-based AMSs. Section 3.2 defines the lifecycle stages of the hailstorm and statistically analyzes data from two weather radars at different stages. Finally, Section 4 provides the conclusions and discussion.

## 2. Materials and Methods

### 2.1. Layout of Observational Equipment for the Severe Precipitation Event Accompanied by Hail

At 05:38:00 UTC on 18 August 2019, the Shanghai central meteorological observatory issued a yellow lightning warning, indicating that lightning activity was expected across most parts of the city within the next six hours, accompanied by short-term heavy precipitation of approximately 20 mm per hour. At 07:10:00 UTC, a Blue Rainstorm Warning was issued, forecasting that cumulative rainfall in the central and southern parts of the city would exceed 50 mm over the next 6 h due to the influence of a strong thunderstorm cloud cluster.

Starting from 07:05:00 UTC, three districts on the western side of the Shanghai administrative region received successive hail warnings. At 07:05:00 UTC, a yellow hail warning was issued for Songjiang District in the southwestern part of Shanghai, predicting that parts of this district would experience hail within the next six hours due to the influence of a strong thunderstorm cloud cluster. At 07:40:00 UTC, a yellow hail warning was issued for Jinshan District, which is adjacent to the southern side of Songjiang District, predicting hail across the district within the next six hours. At 08:42:00 UTC, a Yellow Hail Warning was issued for Jiading District, which is located south of Songjiang District, predicting hail within the next six hours due to the influence of a strong thunderstorm cloud cluster.

**Figure 1:**
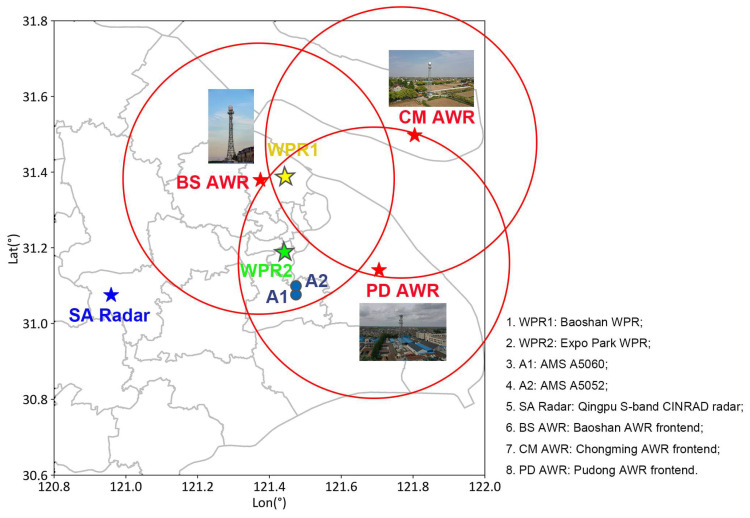
Layout of various meteorological observation equipment in the Shanghai region. Red pentagrams mark the locations of the Shanghai AWR’s three radar frontends, while red circles show their approximate 44 km horizontal detection ranges. The Qingpu SA is labeled “SA Radar” with a blue pentagram. The Baoshan and Expo Park L-band WPRs are labeled WPR1 and WPR2, respectively. Two blue dots mark ground-based AMSs (A5060 and A5052) within the enhanced detection area (EDA) [47], the overlapping range of two AWR frontends**.**

Figure 1 shows the layout and relative positions of various meteorological observation equipment in the Shanghai region. There are two weather radars: the Shanghai AWR and the Qingpu SA radar. The three red pentagrams represent the three radar frontends of the Shanghai AWR, which are arranged in a triangular configuration and deployed in Baoshan District (BS AWR), Pudong District (PD AWR), and Chongming District (CM AWR). The detection range radius of each radar frontend is approximately 44 km, with the red circles representing the horizontal projection of the detection range of each frontend. Table 1 shows the parameters of the Shanghai AWR. The Qingpu SA radar is represented by a blue pentagram in Figure 1. Two L-band boundary-layer WPRs are located at Baoshan National Climate Observatory and Shanghai Expo Park. In addition, the Shanghai Meteorological Bureau has deployed multiple ground-based AMSs to provide conventional ground meteorological data, including air temperature, hourly precipitation, wind direction, and wind speed. Within the overlapping detection area of the Shanghai AWR, the two ground-based AMSs closest to the precipitation cell analyzed in this study are numbered A5060 and A5052 (referred to as A1 and A2 in Figure 1, respectively). These AMSs are represented by two blue dots in Figure 1, showing their positions and spatial relationships with other observation equipment.

### 2.2. Synoptic Analysis and Meteorological Background

**Figure 2:**
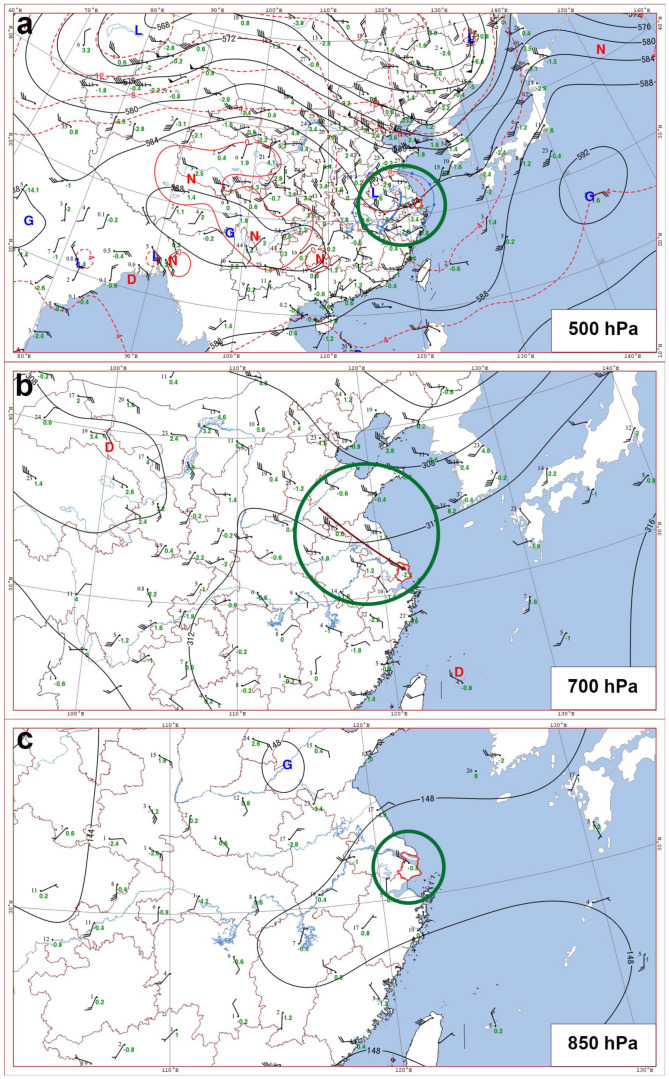
Weather charts at 500 hPa (**a**), 700 hPa (**b**), and 850 hPa (**c**) at 00:00 UTC on 18 August 2019. The red line within the green circle represents the administrative boundary of Shanghai. “G” indicates the high-pressure center, “D” represents the low-pressure center, “L” denotes the cold center, and “N” indicates the warm center. The black contour lines are isobars, and the red contour lines are isotherms. At 500 hPa (**a**), the blue solid line represents the shortwave trough, and the blue circled area indicates the significant cooling region. At 700 hPa (**b**), a brown arrow represents northwesterly airflow. In all three subfigures, the black numbers represent the dew point temperature difference, while the green numbers represent the 24 h temperature change. In the wind barbs, a long feather represents a wind speed of 4 ms^−1^, two long feathers indicate 8 ms^−1^, and a short feather represents 2 ms^−1^, and so on.

From 06:00 to 11:00 UTC on 18 August 2019, persistent precipitation occurred in Shanghai. The synoptic analysis and meteorological background analysis of this precipitation event were conducted using 00:00 UTC sounding data, which was closest in time to the onset of precipitation provided by the Meteorological Information Comprehensive Analysis and Processing System 4.0 (MICAPS 4.0), the Chinese Meteorological Administration’s operational meteorological forecast system. Figure 2 presents weather charts at 500 hPa, 700 hPa, and 850 hPa. The following is an analysis of the three weather charts at these three altitude levels.

In the 500 hPa weather chart at 00:00 UTC on August 18 (Figure 2a), a shortwave trough (blue solid line) can be observed over southern Jiangsu and Anhui provinces, which is expected to continue moving eastward and affect the Shanghai region (the red solid line within the green circle represents the administrative boundary of Shanghai in Figure 2a). Meanwhile, a large negative temperature anomaly exists over Jiangsu, Anhui, Zhejiang, and Hubei Provinces, with a cold center located in central Anhui. The Shanghai region has already experienced a negative temperature anomaly of −5 °C, indicating that the leading edge of the cold air has penetrated the Shanghai region. In the 700 hPa weather chart (Figure 2b), northwesterly airflow (indicated by a brown arrow in Figure 2b) extends from southern Shandong through Jiangsu to Shanghai. The Shanghai region continues to experience a negative temperature anomaly of −2.2 °C, but the dew point temperature difference is 6, indicating poor humidity conditions. In the 850 hPa weather chart (Figure 2c), southwesterly flow can be observed in southwestern Jiangsu, leading to weak wind field convergence. The Shanghai region remains under northwesterly wind with a speed of 8 ms^−1^, and the dew point temperature difference is 9, indicating that the humidity conditions are still poor.

**Figure 3:**
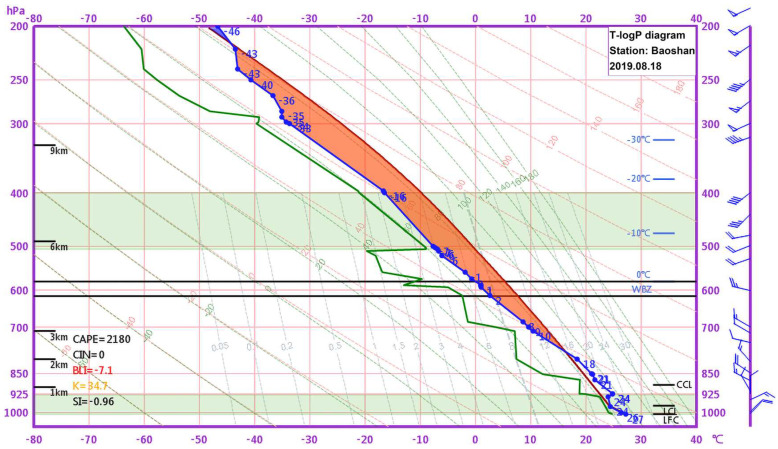
Temperature–pressure logarithmic (T-logP) diagram of the sounding data from the Baoshan Sounding Station in Shanghai at 00:00 UTC on 18 August 2019. The horizontal axis represents temperature (in °C), and the vertical axis represents pressure (in hPa). The wind direction and wind speed at various altitudes are shown on the far right of the diagram. The altitude ranges shaded in light green represent areas with relative humidity ≥ 80%.

The Baoshan National Climate Observatory (represented by the yellow pentagram in Figure 1) is a national primary sounding station in China. The station utilizes a GFE (L) secondary WPR, along with radiosondes and sounding balloons, which are released twice daily at 00:00 and 12:00 UTC for upper-air observations. Figure 3 presents a temperature–pressure logarithmic (T-losgP) diagram derived from the sounding data collected at 00:00 UTC on 18 August 2019, which is the closest in time to the observed precipitation event.

Figure 3 shows a moist layer near the ground in the Shanghai region (between 1000 hPa and 925 hPa). The spacing between the green dew point profile and the blue temperature profile in the near-ground layer is relatively small, indicating favorable humidity conditions. However, in the middle and lower levels (between 600 hPa and 925 hPa), the spacing between the green dew point profile and the blue temperature profile is larger, indicating relatively dry air. In an altitude range of 400 hPa to 500 hPa, humidity conditions are favorable, and the temperature falls between −10 °C and −20 °C. These humidity and temperature conditions are conducive to hail formation. The energy conditions are also favorable, with a convective available potential energy (CAPE) value as high as 2180 J/kg and no convective inhibition (CIN = 0). Based on the instability indices, the K index is 34.7, which is favorable for severe weather development, while the Showalter index (SI) is −0.96, indicating an unstable atmospheric stratification. Once convective triggering conditions occur, a convective precipitation process is highly likely to develop.

**Figure 4:**
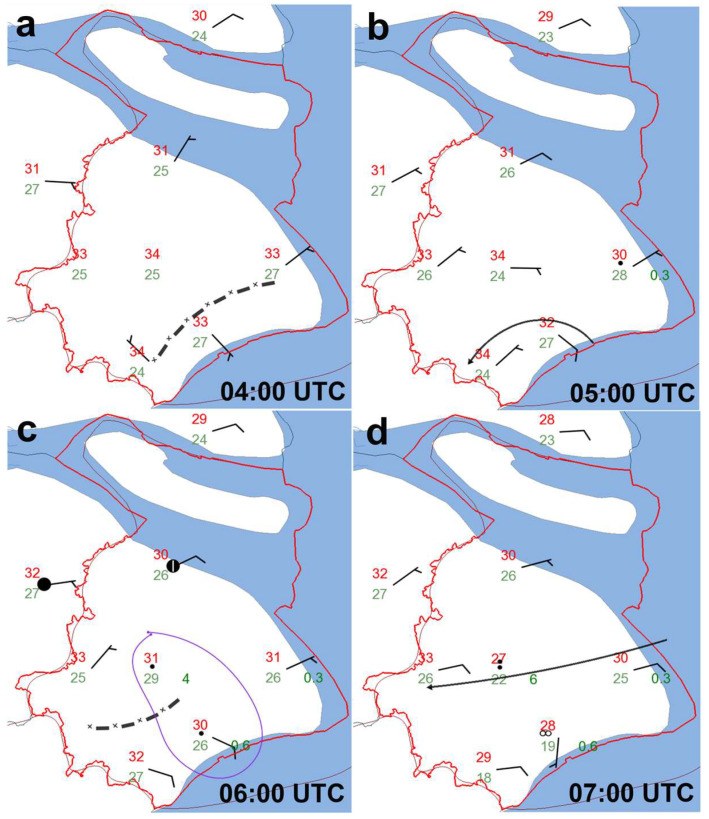
Observations from the ground-based AMSs in the strong precipitation echo region of interest on 18 August 2019. (**a**–**d**) represent observations at 04:00, 05:00, 06:00, and 07:00 UTC, respectively. The red solid line represents the administrative boundary of Shanghai, the green numbers indicate the total precipitation over the past six hours (in mm), the red numbers represent the ground temperature, and the black dashed line indicates the near-ground wind field convergence line. Additionally, wind direction and speed data observed by the ground-based AMSs are provided in the four subfigures.

Figure 4 shows the distribution of ground observation data at different times, as provided by the MICAPS. The ground observation charts show that at 04:00:00 UTC, the temperature across Shanghai generally exceeded 30 °C, particularly reaching up to 34 °C in central and southern areas. At the same time, a ground wind field convergence line was present in the southern region (represented by the black dashed line). At 05:00:00 UTC, the convergence of the wind field remained in the southern region, while precipitation had already started in the southeastern part of the city.

At 06:00:00 UTC, the ground wind field convergence line (black dashed line) moved slightly northward, and precipitation occurred in the southern region (marked by the purple circle). At 07:00:00 UTC, the wind field generally turned into a consistent easterly flow, bringing moisture from the sea, which supported continued precipitation. After 11:00:00 UTC, the precipitation weakened, and the ground wind field was mainly northwesterly to northerly, with no significant wind field convergence lines observed. During the main precipitation event, the maximum cumulative rainfall over a six-hour period in some parts of Shanghai reached up to 33 mm.

**Figure 5:**
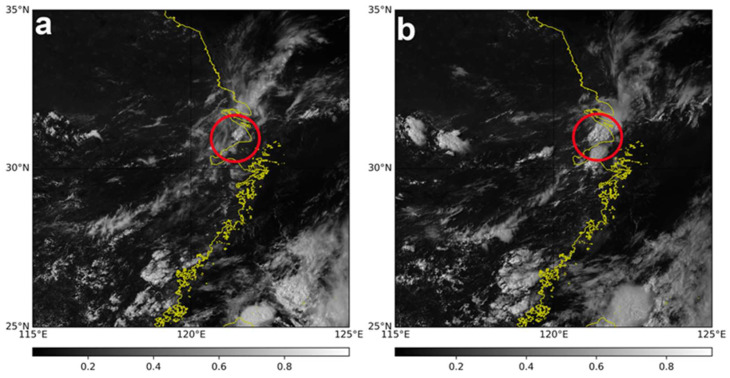
Visible cloud images from Advanced Geostationary Radiation Imager (AGRI) Channel 2 (0.65 μm) on the Fengyun-4 geostationary satellite at two different times on 18 August 2019: 05:42:50 UTC (**a**) and 06:57:50 UTC (**b**). The red circled area indicates the region in Shanghai where the hail event occurred. Over two hours, the cloud system moved in an easterly direction.

To understand the distribution and evolution of the cloud system during the hail event on a larger scale, Figure 5 presents visible cloud images from the Advanced Geostationary Radiation Imager (AGRI) Channel 2 (0.65 μm) onboard the Fengyun-4 (FY-4) geostationary satellite at two different times: 05:42:50 and 06:57:50 UTC. The thicker the clouds, the greater the albedo, the higher the brightness, and the whiter the tone. By comparing Figure 5a,b, referring to multiple FY-4 visible cloud images over approximately two hours (from 05:27:50 to 07:27:50 UTC), it can be seen that most of the area over Shanghai (within the red circle) was covered by clouds. Over the hour following 05:42:50, the cloud body became thicker, taller, and more extensive in coverage, and the movement direction of the cloud system was due east.

## 3. Results

### 3.1. Analysis of WPR and Ground-Based AMS Data

**Figure 6:**
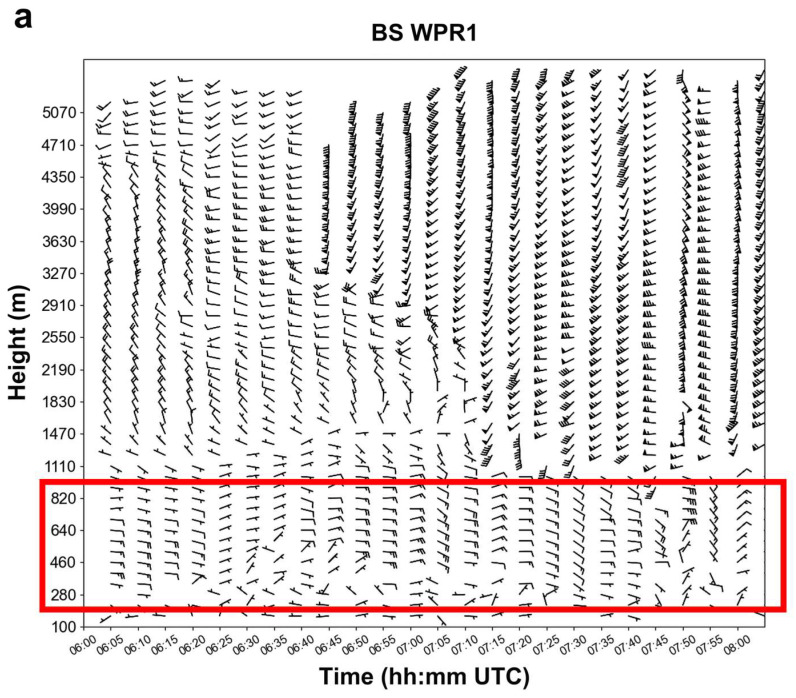

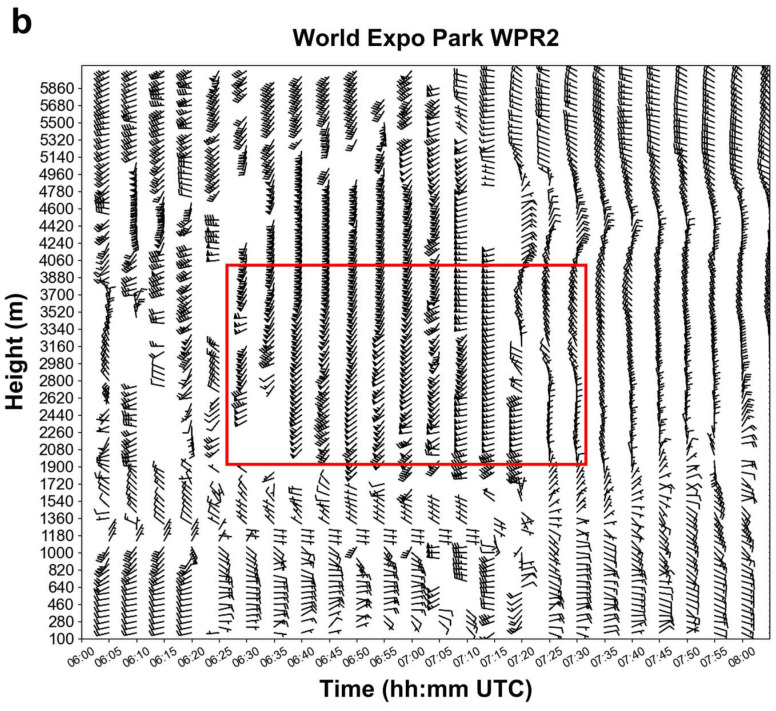
Horizontal wind field detection data at different altitude levels from the L-band WPR1 at the Baoshan Meteorological Observatory (**a**) and WPR2 at Expo Park (**b**) in Shanghai, from 06:00:00 to 08:00:00 UTC on 18 August 2019. The red rectangles in the subfigures highlight the wind data of interest related to the precipitation event. In the wind barbs, a long feather represents a wind speed of 4 ms^−1^, two long feathers indicate 8 ms^−1^, and a short feather represents 2 ms^−1^, and so on.

Figure 6 presents horizontal wind field data at different altitude levels from the L-band WPRs at the Baoshan Meteorological Observatory (WPR1) and the L-band WPR at the Shanghai Expo Park (WPR2) from 06:00 to 08:00 UTC on 18 August 2019. The red rectangle indicates wind direction and wind speed data at different heights during the period of interest.

Figure 1 shows that both WPRs are located within the overlapping detection areas of the Shanghai AWR. The Baoshan Meteorological Observatory is located within a fine detection area (FDA), which is the overlapping detection area of the three phased-array radar frontends of the AWR. WPR2 at Expo Park is located within one of the enhanced detection areas (EDAs), which is the overlapping detection area of two radar frontends, BS AWR and PD AWR.

The convective system associated with hail, which also experienced heavy rainfall, exhibited significant expansion and movement after entering the overlapping detection areas of the Shanghai AWR. The growth of the precipitation event was directed from north to south. Since both WPR1 and WPR2 were located within the AWR’s overlapping detection areas, it was possible to observe the wind field configuration at various altitudes at these two locations during the expansion of the precipitation event (although its overall movement was from west to east, after entering the AWR overlapping detection areas, the precipitation echo expanded from south to north). The Baoshan Meteorological Observatory (31.4° N, 121.4° E, where WPR1 is located) is situated in northwestern Shanghai, approximately 7.5 km away from the nearest BS AWR, and the heavy precipitation event was located more than 7.5 km away.

The wind data from WPR1 during the precipitation period from 06:30:00 to 07:30:00 UTC (Figure 6a) provide valuable insights into the dynamics and moisture transport associated with the severe precipitation event. In Figure 6a, below 0.3 km, the wind direction was relatively disordered, with wind speeds mostly around 2 ms^−1^. In an altitude range of 0.3 km to 0.8 km, there was a predominantly easterly flow, indicating moisture transport from the direction of the Yangtze River estuary, with wind speeds mostly ranging from 4 to 8 ms^−1^. Above 1 km, there was a significant change around 06:40:00 UTC: before 06:40:00, the wind was predominantly northwesterly, while after 06:40:00, strong southwesterly winds began to descend from above 3 km, with wind speeds exceeding 20 ms^−1^ at times.

The wind data from WPR2 In the Expo Park area (Figure 6b) cover the period from 06:00:00 to 08:00:00 UTC. Between 06:00:00 and 06:20:00, the wind at a 1 km altitude was predominantly southwesterly, with relatively high wind speeds, exceeding 10 ms^−1^ in some areas. From 06:20:00 to 07:05:00, during a 45 min period, easterly winds dominated at a 1 km altitude, indicating moisture transport from the Yangtze River estuary. Between 07:05:00 and 07:20:00, the wind shifted to southwesterly–northwesterly. Finally, from 07:25:00 to 08:00:00, during a 35 min period, easterly winds returned to dominate at a 1 km altitude. This shows that during the precipitation period of interest (06:00:00 to 07:30:00 UTC), easterly winds dominated the lower levels most of the time, providing moisture transport for the event. From 06:25:00 to 07:20:00, at altitudes above 1.9 km (within the red rectangle in Figure 6b), strong southwesterly winds appeared, with wind speeds mostly exceeding 20 ms^−1^. This indicates substantial energy in the storm, suggesting sufficient dynamic transport. Through statistical analysis of the Shanghai AWR data in Section 3.2.2, it is evident that during this nearly 1 h period, the precipitation event underwent the development, maturity, and dissipation stages.

**Figure 7:**
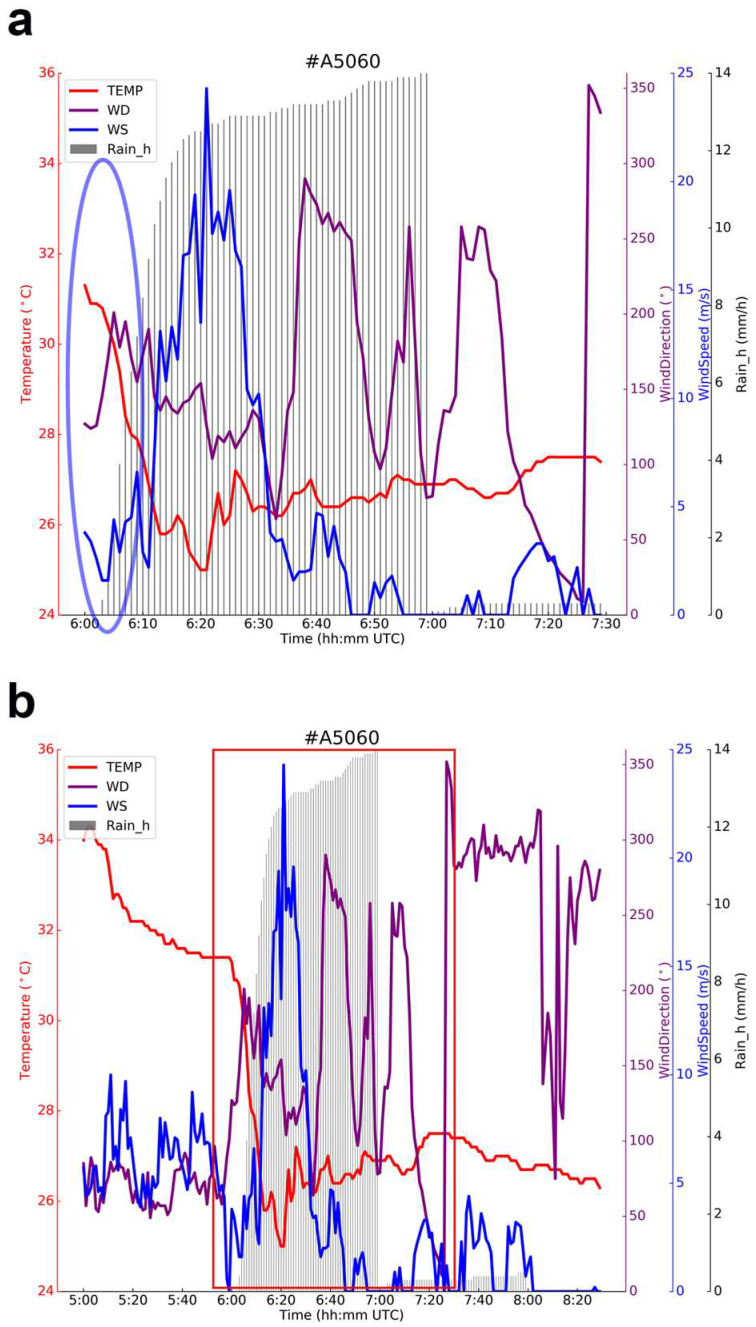

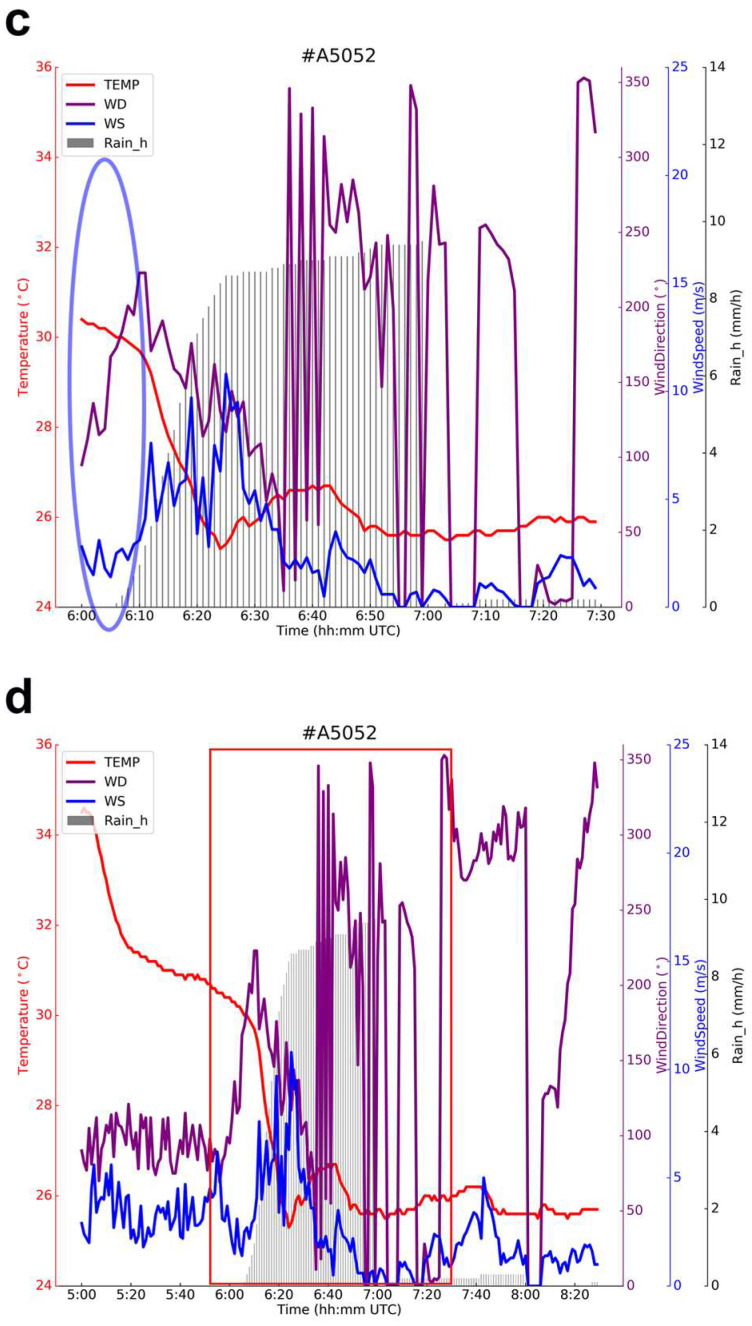
Ground hourly precipitation, wind direction, wind speed, and temperature data obtained from two AMSs, A1 ((**a**,**b**) A5060 station) and A2 ((**c**,**d**) A5052 station), within a fine detection area (FDA) [47] of Shanghai AWR during the precipitation period from 05:00:00 to 08:30:00 UTC on 18 August 2019. The blue circles indicate time range where changes in wind direction are of particular interest, while the red boxes (**b,d**) highlight the main precipitation periods, which are shown in greater detail in **a** and **c**. The relative positions of A1 and A2, as well as their layout with respect to other observation equipment, can be referenced in Figure 1.

According to the AWS data shown in Figure 7a,d, between 05:00:00 and 08:30:00, data from two AWSs within the overlapping detection areas of the Shanghai AWR, A5060 and A5052 (labeled A1 and A2 in Figure 1), indicate that the peak rainfall for both stations occurred between 06:00:00 and 07:00:00, as shown in Figure 7a,c, with hourly precipitation reaching 14 mm and 9 mm, respectively. Following the onset of precipitation, the ground temperature at both stations immediately dropped from over 34 °C at 05:00:00 to a low of around 25 °C by about 06:20:00. As shown in Figure 1, stations A5060 and A5052 are very close to each other, with A5060 situated south of A5052. The analysis in Section 2 shows that during the focused precipitation period (06:00:00 to 07:30:00, as shown in Figure 7a,c), the direction of precipitation cell radar echo expansion was from south to north, reaching station A5060 before station A5052.

After the precipitation cell reached the station’s ground at 06:02:00, the ground wind speed at the station increased sharply. The instantaneous wind speed reached a maximum value of 25 ms^−1^ 21 min later (at 06:23:00), meeting the definition of a “strong wind”. Subsequently, the wind speed gradually decreased. Figure 7a is an enlarged view of the data within the red box in Figure 7b. The hourly rainfall data presented in Figure 7a show that the rainfall intensity increased significantly from 06:02:00 to 06:23:00 (indicated by the gray bar chart in the red box). The amount of rainfall reached 12 mm in just 21 min. From 06:23:00 to 07:00:00, however, only about 2 mm of rain fell over this 37 min period. From 07:00:00 to 07:30:00, the amount of rainfall was less than 2 mm.

During the main precipitation period at station A5060 (06:02:00–07:00:00), the ground wind direction changed frequently. Overall, in the first half of the period, the wind was predominantly from the southwest and southeast (indicated by the blue circle in Figure 7b during the initial phase of precipitation, which coincided with the expansion direction of the precipitation echo). In the second half of the period, the wind predominantly shifted to the southeast and northwest. Combined with the ground rainfall data from the station, the rapid increase in wind speed after the precipitation reached the ground at 06:02:00 is consistent with strong ground winds caused by intense downdraft divergence in convective storms.

Due to the south-to-north expansion direction of the precipitation event radar echo, station A5052 was affected by the precipitation event later than station A5060. Figure 7c shows that the precipitation reached station A5052 at 06:05:00, 3 min later than at station A5060. After the precipitation cell reached the station, the temperature rapidly dropped to below 26 °C from above 30 °C, and the wind speed peaked at 10 ms^−1^ at 06:25:00. The gradients of the temperature decrease and wind speed increase at station A5052 were smaller than that at station A5060, indicating a loss of energy in the precipitation cell. During the main precipitation period (06:05:00–07:00:00), the wind speed varied between 0 and 10 ms^−1^, and the wind direction changed sequentially from southwest to south to southeast (indicated by the blue circle in Figure 7c, where the wind direction aligned with the direction of the expanding precipitation event at the beginning of the precipitation). From 06:35:00 onward, the wind direction underwent a drastic change. Similar to the relationship between Figure 7a,b, Figure 7c represents a magnified view of the area outlined by the red box in Figure 7d.

### 3.2. Statistical Analysis of Data from Two Weather Radars

#### 3.2.1. Classification of the Three Lifecycle Stages of the Strong Convective Precipitation Process

Due to the high-spatiotemporal-resolution data provided by the three phased-array frontend AWR in Shanghai and the ability to perform wind field retrieval in the overlapping detection areas, this study primarily focuses on the data from the Shanghai AWR to analyze the severe precipitation event associated with hail, with supplementary data from the Qingpu SA radar.

**Figure 8:**
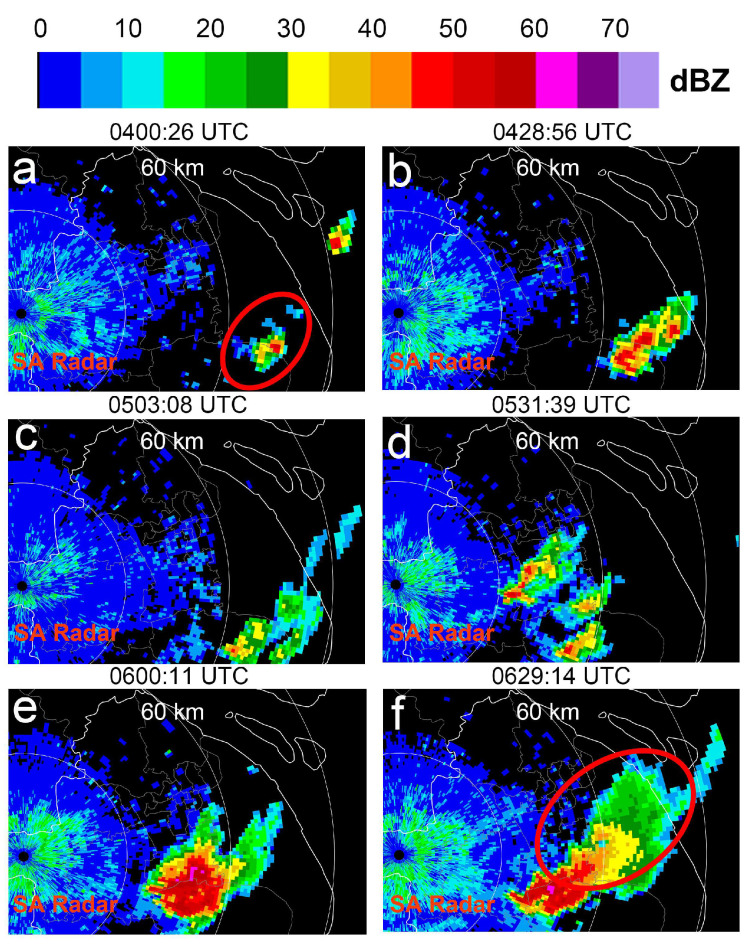
Reflectivity PPI images of precipitation event detected by Qingpu SA radar at an elevation angle of 2.37° from 04:00:26 to 06:29:14 UTC on 18 August 2019, with a focus on the development of radar echoes, highlighted by the red ellipses. At 04:00, a small precipitation echo appeared approximately 70 km to the southeast of the Qingpu SA radar (within the red ellipse (**a**). Over the following two hours, this echo gradually intensified (**a**–**e**). At one point, it split into three relatively independent echo cells (**d**) By 06:00:11 (as shown in Figure **e**), the three echo cells merged into a new and larger echo. The precipitation process continued to develop and expanded further toward the northeast of the radar (**f**). This precipitation event entered the overlapping detection areas of the Shanghai AWR around 06:00. This study focuses on analyzing high-spatiotemporal-resolution data from the Shanghai AWR for approximately 90 min after 06:00 when the precipitation radar event entered the AWR overlapping detection areas.

During the precipitation period of interest, 06:00:00 to 07:30:00 UTC, the Shanghai AWR detected a severe precipitation event associated with hail. As the radar’s geographic location is fixed, the precipitation event had already been developing before the period of interest, and after reaching a certain stage, the precipitation cell entered the overlapping detection areas of the Shanghai AWR. To understand the development of the precipitation cell before the time of interest, Figure 8 provides a series of reflectivity plan position indicator (PPI) images from the Qingpu SA radar at an elevation angle of 2.3°, taken approximately every 30 min. Figure 8a–f show that at 04:00:00, a small precipitation radar echo appeared around 70 km southeast of the Qingpu SA radar (indicated by the red circle in Figure 8a). This precipitation cell gradually strengthened and expanded and then split into three relatively independent small echo clusters (Figure 8d). At 06:00:11 (Figure 8e), the three small precipitation echoes merged to form a new precipitation echo, which expanded slightly to the northeast, with part of the northern precipitation echo beginning to enter the overlapping detection areas of the Shanghai AWR. The last subfigure, Figure 8f, shows that the precipitation process continued to develop and further expanded to the northeast.

**Figure 9:**
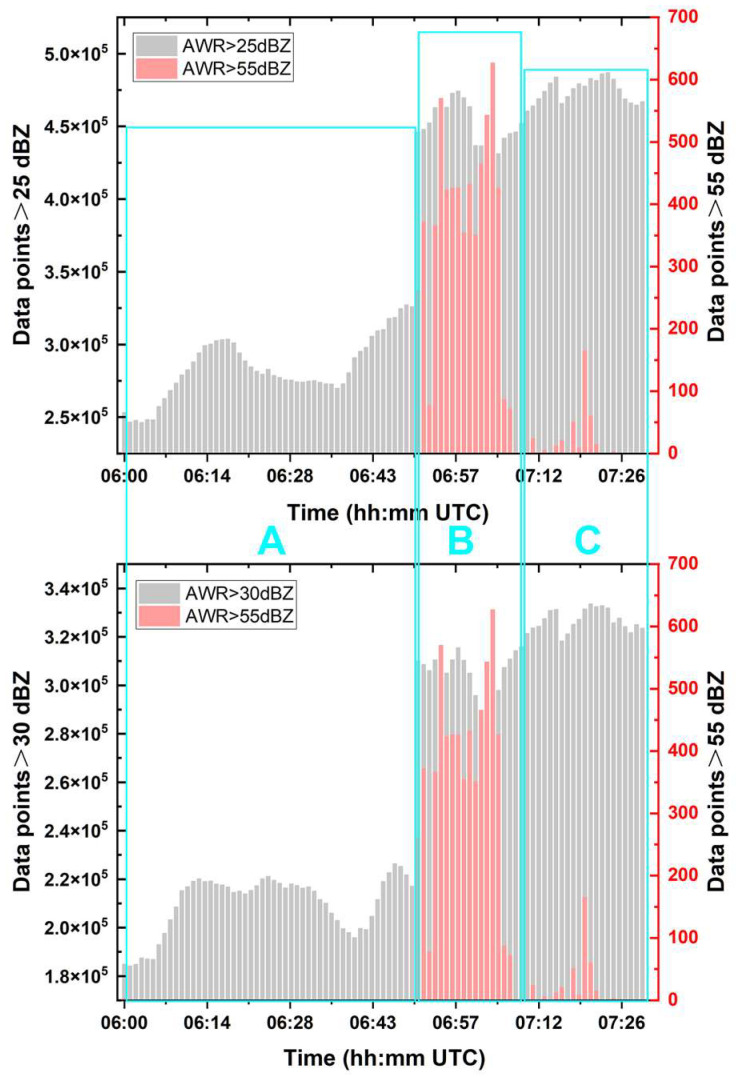
Temporal variation trends of statistical data for different reflectivity factor thresholds of the precipitation echo of interest detected by the Shanghai AWR on 18 August 2019. The figures also illustrate the classification of the three lifecycle stages of the event based on the statistical results using different reflectivity factor thresholds. Three green boxes labeled A, B, and C represent the initial development stage, the maturity stage, and the dissipation stage of the precipitation cell of interest in this study, respectively.

Since the Shanghai AWR is a phased-array radar with three phased-array frontends, it provides higher spatiotemporal-resolution data than the SA radar, allowing for more detailed information on the evolution of precipitation cloud systems. Before analyzing the observational data captured by the AWR during the precipitation event, the lifecycle of the precipitation cell during the period of interest (06:00:00–07:30:00) was defined. After counting the number of data points with reflectivity values greater than 25 dBZ, 30 dBZ, and 55 dBZ within the Shanghai AWR overlapping detection areas, we defined the primary period when the reflectivity factor exceeded 55 dBZ as the mature stage of the precipitation cell’s lifecycle. Additionally, the period before the mature stage, during which reflectivity factors exceeding 25 dBZ were most prevalent, is defined as the development stage, while the stage during which the data points with reflectivity values greater than 55 dBZ rapidly disappear is defined as the dissipation stage. In other words, the precipitation process is divided into three stages: the development stage (stage A, before 06:50:00), the mature stage (stage B, 06:51:00–07:07:00), and the dissipation stage (stage C, after 07:08:00). These correspond to the time intervals highlighted by the cyan boxes (A, B, and C) in Figure 9.

**Figure 10:**
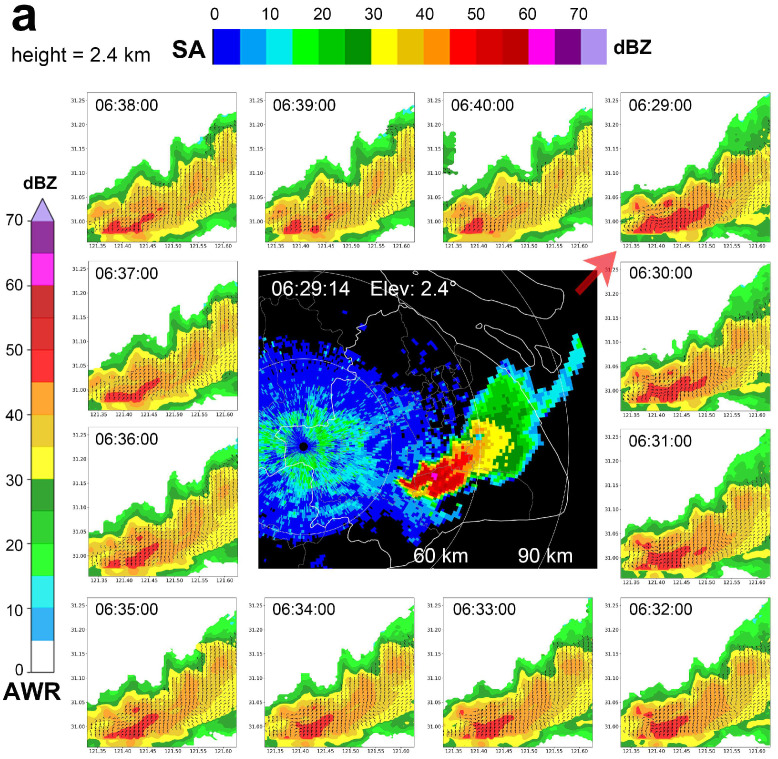

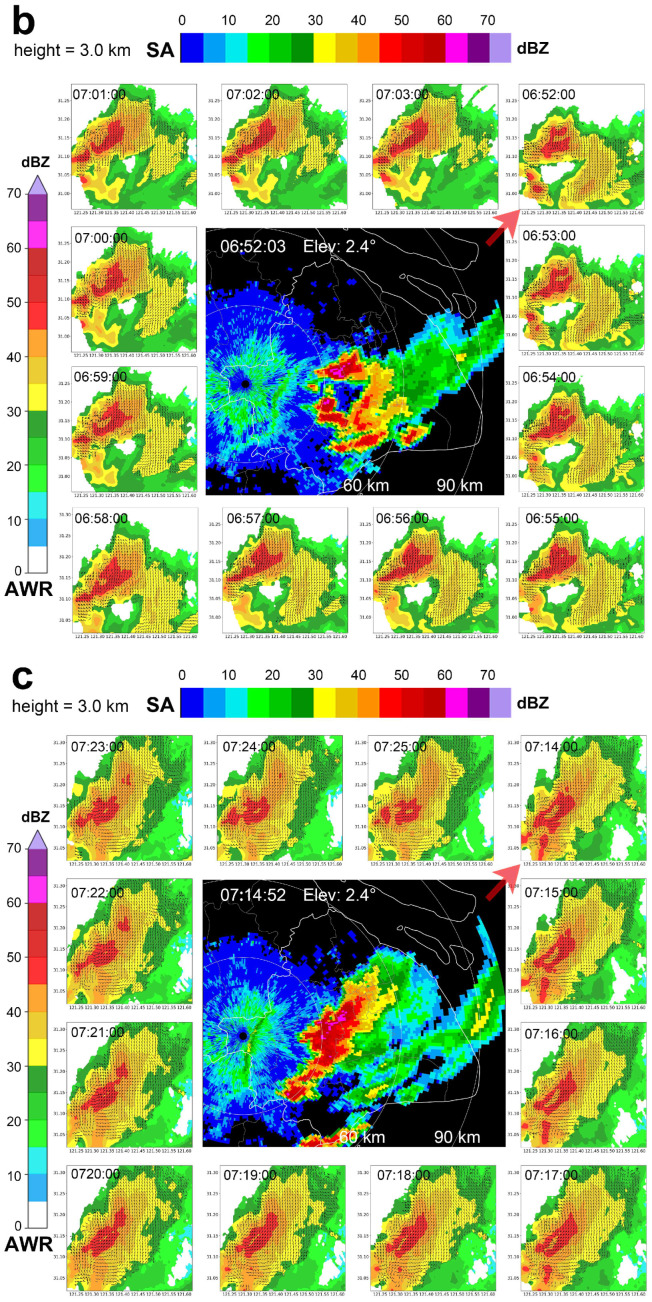
Observational data for the convective precipitation cell of interest detected by the Shanghai AWR and the Qingpu SA radar at three different stages of its lifecycle on 18 August 2019: (**a**) development; (**b**) maturity; (**c**) dissipation. In each subfigure, the PPI figure with a black background at the center shows the reflectivity factor data detected by the Qingpu SA radar. Surrounding the SA radar data, the small figures with white backgrounds display the reflectivity factor data overlaid with wind field data from the Shanghai AWR. For each of the three stages, the small subfigure at the upper right of each subfigure which is pointed out by a red arrow represents the starting time, and AWR observational data figures are shown at 1 min intervals thereafter.

To demonstrate the advantages and detection capabilities of the AWR, Figure 10a–c present continuous 12 min observations for three distinct stages of the precipitation lifecycle, based on the classification results of precipitation echo lifecycle stages from the statistical analysis of the Shanghai AWR observations shown in Figure 9. Specifically, Figure 10a represents the developing stage, Figure 10b represents the mature stage, and Figure 10c represents the dissipating stage. In each subplot, reflectivity factor images superimposed with wind field data from the AWR overlapped detection areas are shown against a white background, whereas reflectivity factor images from the Qingpu SA radar are shown against a black background. The volume scan update interval is 6 min for the Qingpu SA radar and 30 s for the Shanghai AWR. In other words, while the Qingpu SA radar completes one volume scan, the Shanghai AWR can complete twelve.

Due to length constraints and to display observational data over a longer duration, Figure 10a–c are arranged such that, starting from the first subplot in the upper right corner of each subfigure, the Shanghai AWR data are displayed sequentially at 1 min intervals in a clockwise manner. At the center of each subplot is one reflectivity factor image from the Qingpu SA radar. Although the wind field retrieval and wind field validation capabilities of the AWR have been previously evaluated [43,44,45,47,49,50,51], the continuous Shanghai AWR data presented in Figure 10a–c further illustrate its ability to continuously detect reflectivity intensity fields and retrieve wind fields within overlapped detection areas.

Moreover, Figure 10a–c confirm the high spatial-resolution detection capabilities of the Shanghai AWR. Due to the Qingpu SA radar’s radial range resolution (1 km), its reflectivity factor data, displayed against a black background, clearly show a mosaic-like, blocky structure. By contrast, as described in Table 1, the Shanghai AWR observational data grid resolution is 0.1 km × 0.1 km × 0.1 km, and the reflectivity factor data, displayed against a white background, are evidently finer than the Qingpu SA radar data. Subsequently, wind field products from the AWR will be used to further analyze this precipitation event.

**Figure 11:**
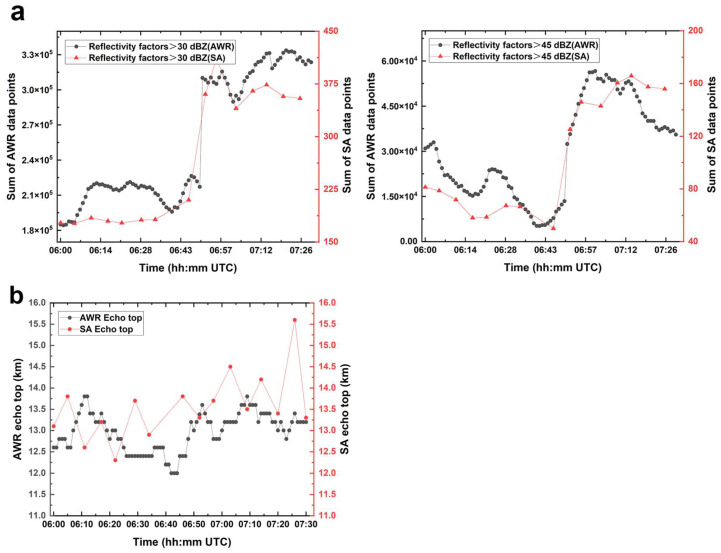
Statistical analysis and comparison of convective cell reflectivity factor data points (**a**), as well as statistical analysis and comparison of echo top (ET) products (**b**), derived from Qingpu SA radar and Shanghai AWR observations on 18 August 2019.

Figure 11a presents a comparative statistical analysis of the number of data points exceeding reflectivity factors of 30 and 45 dBZ from the Qingpu SA radar and the Shanghai AWR, per the precipitation echo illustrated in Figure 10. In Figure 11a, red triangles represent the statistical results derived from Qingpu SA radar volume scans, while black dots represent the statistical results from Shanghai AWR volume scans. The volumetric scanning data from the Shanghai AWR provide more detailed insights into the temporal variations in echo intensity. For data points exceeding 30 dBZ, although both radars exhibit a generally oscillatory increase followed by a gradual decline, the total AWR data points exhibit seven distinct peaks, whereas the SA radar data show a smoother pattern. In the data points exceeding 45 dBZ, an oscillatory increase is also evident, but the oscillations recorded by the Shanghai AWR are more obvious. During the dissipation phase, the number of high-reflectivity data points detected by the Shanghai AWR decreases more rapidly than those detected by the SA radar.

Figure 11b shows temporal variation in the echo top (ET) of the precipitation echo of interest, as detected by both the Qingpu SA radar and the Shanghai AWR. With its higher temporal resolution, the ET product derived from the Shanghai AWR provides more detailed information and exhibits a relatively smooth trend. By contrast, the ET product from the SA radar demonstrates more abrupt fluctuations.

In the following section, we will use the high-spatiotemporal-resolution detection data from the Shanghai AWR to perform a more detailed analysis of the three stages of this strong convective precipitation cell (development, maturity, and dissipation).

#### 3.2.2. Analysis of the Strong Convective Precipitation Process Accompanied by Hail Using Shanghai AWR Detection Data

The strong precipitation echo region of interest entered the overlap detection areas of the Shanghai AWR during the focus precipitation period (06:00:00–07:30:00). This section provides a detailed analysis of the intensity, divergence, and vertical vorticity components of this strong convective precipitation process.

**Figure 12:**
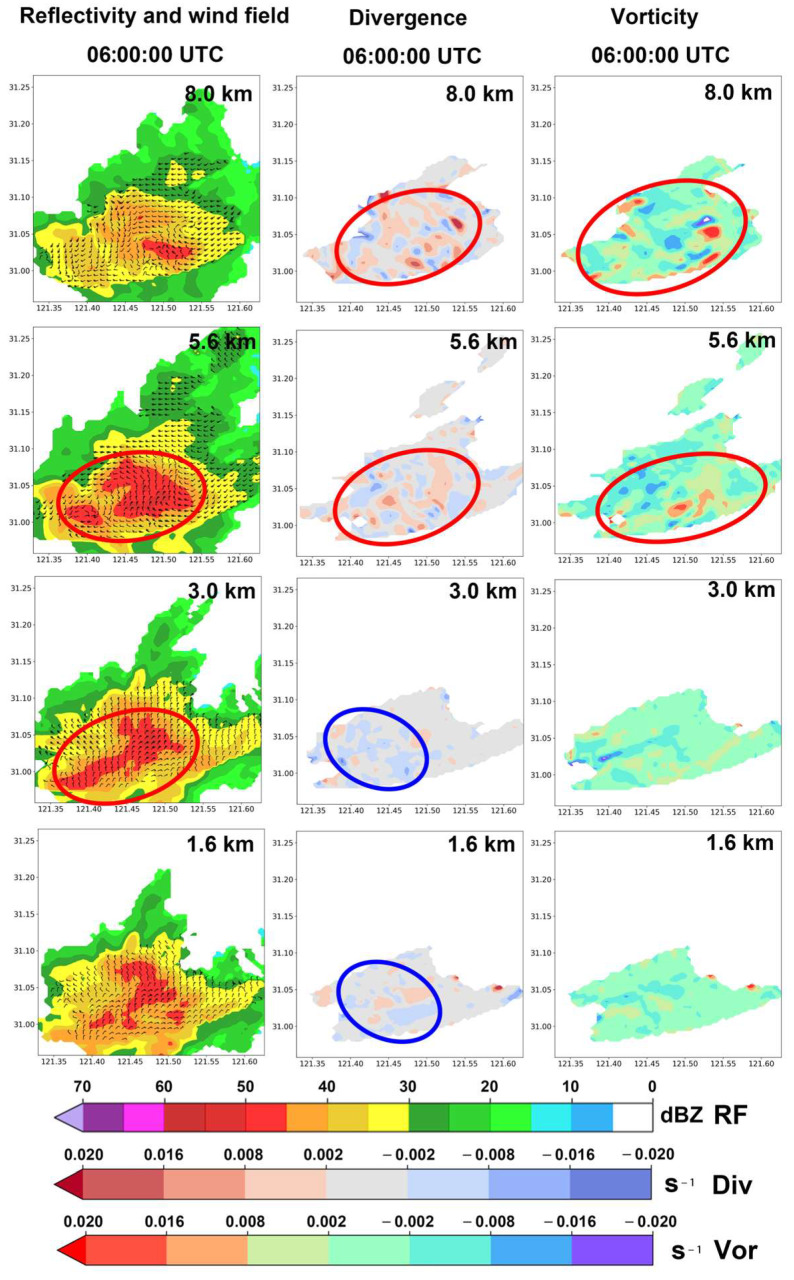
Data from the Shanghai AWR on 18 August 2019, showing the convective precipitation cell of interest during its development stage at 06:00:00 UTC at various heights: (1) reflectivity factors combined with the wind field (left column); (2) horizontal divergence (middle column) calculated using the wind field from the AWR; and (3) vertical vorticity components (right column) calculated from the AWR wind field. The red ellipses and the blue ellipses indicate the areas of particular interest, respectively.

The reflectivity factor plots at each altitude in the left column of Figure 12 show that during the development stage of the precipitation echo, red regions with reflectivity factors exceeding 45 dBZ appear at various altitudes. Overall, the areas with high reflectivity factors in the upper and lower levels are relatively scattered, while the areas with high reflectivity factors at altitudes of 3.0 km and 5.6 km are somewhat more concentrated (highlighted by red ellipses in the left column of Figure 12). This indicates that the kinetic energy at all altitudes of the precipitation cell is sufficient during the development stage.

The divergence fields at each altitude in the middle column of Figure 12 show that at an altitude of 1.6 km, a large negative divergence (horizontal convergence) is present. At 3.0 km, although the negative divergence areas are more scattered and fragmented, there are significant areas of negative divergence (highlighted by blue ellipses in the subfigures). At 5.6 km and 8.0 km, positive divergence and high-value regions dominate, indicating that the water particles in the upper levels of the convective precipitation cell are primarily experiencing divergence. This horizontal divergence in the airflow creates a suction effect, which is conducive to the further development of convection (highlighted by red ellipses in the subfigures).

The right column of Figure 12 shows that the vertical vorticity component fields in the lower levels are relatively balanced between positive and negative values. However, at altitudes of 5.6 km and 8.0 km, these components are predominantly positive, with significant high-value centers of positive vorticity appearing. This suggests strong rotating updrafts in the upper levels of the convective precipitation cell. Additionally, by comparing multiple subfigures in the left and right columns of Figure 12, we can see that at altitudes of 5.8 km and 8.0 km, areas corresponding to the cores of strong precipitation echoes (reflectivity factors > 45 dBZ) in the left column also exhibit large regions of positive vorticity in the right column.

**Figure 13:**
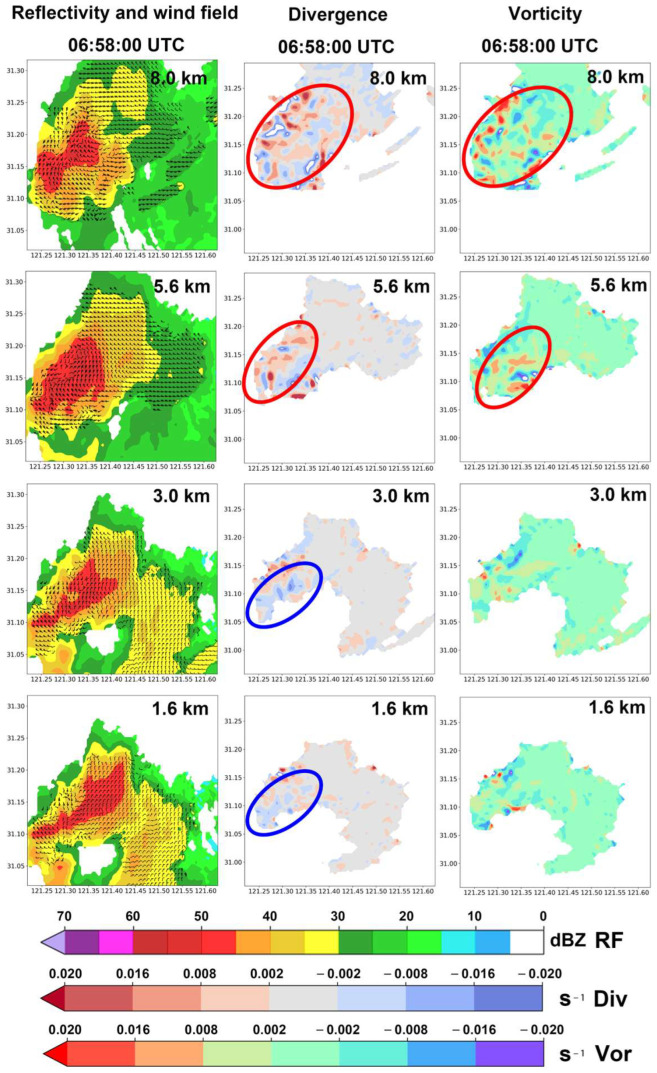
Data from the Shanghai AWR on 18 August 2019, for the convective precipitation cell of interest during its mature stage at 06:58:00 UTC at various altitudes: (1) reflectivity factor overlaid with wind field data (left column); (2) horizontal divergence field derived from the AWR wind field data (middle column); and (3) vertical vorticity component field derived from the AWR wind field data (right column). The red ellipses and the blue ellipses indicate the areas of particular interest, respectively.

Figure 13 presents the reflectivity factor overlaid with wind field data (left column), divergence field data (middle column), and vertical vorticity component field data (right column) at different altitudes for the precipitation echo during the mature stage (stage B) of its lifecycle at 06:58:00 UTC, approximately one hour later.

Compared with the physical quantity data during the development stage of the precipitation echo (Figure 12), during the mature stage, the precipitation echo in the left column of Figure 13 significantly increases, indicating an expansion of the precipitation area. At various altitudes, the areas with reflectivity factors of >45 dBZ in the strong precipitation echo cores have converged and expanded, indicating an increase in rainfall intensity. Specifically, the area with high reflectivity factors at an altitude of 5.6 km is the largest, indicating that at this moment, the primary distribution area of the precipitation core is at a 5.6 km altitude. Since the formation of hail requires sufficient time and vertical space, the comparison in Figure 11b shows that during the precipitation period of interest, ET height remained above 10 km, indicating that the precipitation storm system had a relatively high altitude, providing favorable spatial conditions for hail formation.

The divergence fields at each altitude in the middle column of Figure 13 show that at altitudes between 1.6 km and 5.6 km, there are regions of negative divergence, especially evident at 1.6 km and 3.0 km (highlighted by blue ellipses), indicating horizontal convergence in the mid-to-lower levels of the precipitation cell. At the upper level (8.0 km), positive divergence and regions of high positive divergence values have increased (highlighted by red ellipses), indicating that the upper levels of the convective storm are dominated by divergence; conversely, the high levels of the precipitation cell exhibit divergence, and the mid-to-lower levels exhibit convergence, maintaining the suction effect and ensuring smooth dynamical transmission within the precipitation cell.

The vertical vorticity component fields in the right column of Figure 13 show that compared with the vertical vorticity component in the right column of Figure 12 during the development stage, the high vertical vorticity centers still exist at mid-to-high altitudes (5.6 km–8.0 km). However, they no longer have large continuous areas and have become fragmented (highlighted by red ellipses). This indicates that the rotational updraft dynamics within the mid-to-high levels of the convective storm still exist, but the consistency of dynamical forces among the precipitation particles is weaker than during the initial development stage of the precipitation echo lifecycle.

Additionally, similar to Figure 12, in an altitude range of 3.0 km–8.0 km, the strong precipitation echo cores with reflectivity factors of >45 dBZ in the left column of Figure 13 generally correspond to large regions of cyclonic vorticity (positive vorticity) in the right column.

**Figure 14:**
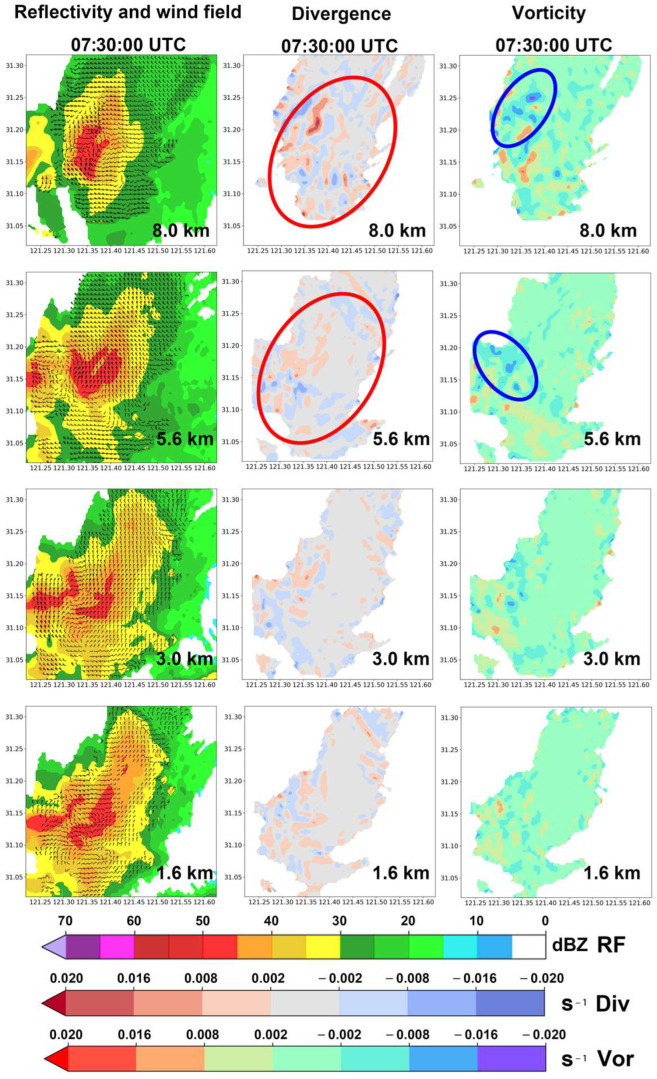
Data from the Shanghai AWR on 18 August 2019, for the convective precipitation cell of interest during its dissipation stage at 07:30:00 UTC at various altitudes: (1) reflectivity factor overlaid with wind field data (left column); (2) horizontal divergence field derived from the AWR wind field data (middle column); and (3) vertical vorticity component field derived from the AWR wind field data (right column). The red ellipses and the blue ellipses indicate the areas of particular interest, respectively.

Approximately 30 min later at 07:30:00 UTC during the dissipation phase (stage C), Figure 14 shows the reflectivity factor overlaid with the wind field data (left column), divergence field data (middle column), and vertical vorticity component field data (right column) at different altitudes.

Compared with the figures in the left column of Figure 13, the strong precipitation core area with a reflectivity factor of >45 dBZ has significantly decreased at all altitudes, indicating that the precipitation echo has entered the dissipation phase at this time.

The divergence field diagrams in the middle column of Figure 14 show that while convergence in airflow still exists at each altitude, the divergence area has notably increased compared with the diagram in Figure 13, taken 32 min earlier. At the 5.6 km and 8.0 km altitudes, the intense divergence has noticeably decreased (the areas with large divergence values are marked with red ellipses), indicating that the upward airflows in the upper layers have weakened.

Compared with the vertical vorticity component fields at different altitudes in the right column of Figure 13, the vertical vorticity field diagrams at each altitude in the right column of Figure 14 show that the large cyclonic vorticity regions at the 5.6 km and 8.0 km mid and high levels have significantly decreased. Meanwhile, anticyclonic vorticity still exists (the large negative vorticity areas are marked with blue ellipses), indicating downward rotating airflows within the mid and high levels of the precipitation cell. These changes in the data characteristics are consistent with the expected physical and dynamic features of the airflow during the dissipation phase of the convective precipitation.

At this stage, although large vorticity areas appear at the same altitude as the high-reflectivity-factor regions, their locations do not show clear correspondence at the same height.

## 4. Conclusions and Discussions

This study utilizes multisource observational datasets to analyze a severe convective precipitation event accompanied by hail that occurred in Shanghai on 18 August 2019. Special emphasis was placed on the high-spatiotemporal-resolution precipitation reflectivity and wind field data provided by a novel X-band AWR within its overlapping detection areas from 06:00 to 07:30 UTC.

Radiosonde analysis indicated favorable conditions for intense convection prior to precipitation onset, characterized by high CAPE, 2180 J/kg, significantly unstable atmospheric stratification, and conducive humidity and temperature profiles.

Integrated analysis of two L-band WPRs—WPR1 at Baoshan Station and WPR2 at Shanghai Expo Park—revealed that the convective system expanded from south to north, supported by moisture transported by easterly winds at low levels and strong southwesterly winds (exceeding 20 m s^−1^) at middle-to-upper levels. Although satellite observations indicated a general storm movement from west to east, local wind field data showed that the expansion pattern corresponded well with the sequential timing of the strong southwesterly wind observed by the two WPRs, with WPR2 detecting the southwest wind approximately 15 min earlier than WPR1. This suggested that the strong southwesterly airflow was likely entrained by the precipitation itself.

Observations from two ground-based AMSs (A5060 and A5052) further confirmed that the precipitation expansion matched local ground wind changes. Upon precipitation arrival, abrupt temperature decreases and rapid wind speed increases occurred at both stations, with station A5060 experiencing a peak wind speed of 25 m s^−1^, approximately 21 min after precipitation onset.

Compared with the Qingpu SA radar’s approximately 6 min volume-scan interval, the rapid-scanning Shanghai AWR provided enhanced high-spatiotemporal-resolution data, offering superior observational conditions for investigating the mechanisms of short-duration severe convective precipitation and facilitating integrated analysis through multisource datasets.

The high-resolution rapid-scan capabilities of the AWR system enabled detailed observations of the precipitation structures and associated wind fields. Utilizing these wind observations, horizontal divergence and vertical vorticity fields at various altitudes were calculated, effectively revealing airflow characteristics such as convergence, divergence, and rotation throughout different storm stages. During the developing and mature stages, clear convergence was observed in the mid-to-lower atmosphere (1.6–3.0 km), accompanied by divergence and prominent cyclonic rotation in the upper levels (5.6–8.0 km). This indicated robust rotating updrafts and dynamic structures supporting severe convective precipitation. However, in the dissipation stage, regions of intense reflectivity (>45 dBZ) diminished significantly, along with a marked reduction in both divergence and cyclonic vorticity, suggesting a weakening of the storm’s updraft mechanisms and rotational characteristics.

Although the X-band AWR radar utilized in this study provided valuable high-resolution observations, its lack of polarimetric capabilities prevented an analysis of dual-polarization parameters, such as differential reflectivity (Z_DR_). Moreover, attenuation effects on the X-band radar data from strong convective precipitation, though significant, were not addressed, as data quality control and attenuation correction are handled separately by specialized researchers.

In future work, we plan to continue research into phased-array weather radar technology, including addressing the critical issues of radar attenuation correction and integrating polarimetric capabilities. By incorporating multisource observational data, such as satellite remote sensing, numerical simulations, and polarimetric radar observations, we aim to achieve deeper insights into microphysical processes and dynamic structures associated with severe convective weather events.

## Figures and Tables

**Table 1:** Specifications of Shanghai AWR.

Array Weather Radar (AWR)	Parameters
Technology	Distributed and active phased array One-dimensional, Doppler, single-polarized
Frequency	9.3–9.5 GHz
Volume scan update time	30 s
Each frontend scanning mode	Mechanical scan horizontally and electronic scan vertically
Maximum detection range of one radar	~44 km
Grid size of outputs	0.1 km × 0.1 km × 0.1 km

## Data Availability

All meteorological data in this article belong to the China Meteorological Administration. If necessary, please contact the Information Center of the China Meteorological Administration for data questions.

## Abbreviations

The following abbreviations are used in this manuscript:

PAR	Phased-array radar
NWRT	National Weather Radar Testbed
LMA	Lightning mapping array
PARISE	Phased-Array Radar Innovative Sensing Experiment
MWR-05XP	Meteorological Weather Radar 2005 X-band Phased Array
WSR-88D	Weather Surveillance Radar-1988 Doppler
S-PAR	S-band vehicle-mounted one-dimensional phased-array weather radar
APAR	X-band dual-polarization phased-array weather radar
C-PAR	C-band phased-array radar
AWR	Array weather radar
SAS	Synchronized azimuthal scanning
DTD	Detection data time difference
BS AWR	Baoshan Array Weather Radar
PD AWR	Pudong Array Weather Radar
CM AWR	Chongming Array Weather Radar
T-logP	Temperature–pressure logarithmic
CAPE	Convective available potential energy
CIN	Convective inhibition
SI	Showalter index
MICAPS	Meteorological Information Comprehensive Analysis and Processing System
AGRI	Advanced Geostationary Radiation Imager
FY-4	Fengyun-4
WPR	Wind profiler radar
FDA	Fine detection area
EDA	Enhanced detection area
PPT	Plan position indicator
ET	Echo top
